# Tumeur pseudopapillaire et solide du pancréas: une cause rare de masse abdominale

**DOI:** 10.11604/pamj.2015.22.361.7390

**Published:** 2015-12-14

**Authors:** Samir Hasbi, Mohammed Menfaa, Fouad Sakit, Jamal Laaroussi, Abdelkrim Choho

**Affiliations:** 1 Service de Chirurgie Viscérale, Hôpital Militaire Moulay Ismaïl, Meknès, Maroc

**Keywords:** Pancréas, tumeur pseudopapillaire et solide, Pancreas, pseudopapillary and solid tumor

## Abstract

La tumeur pseudopapillaire et solide du pancréas (TPPSP) est une tumeur rare, c'est une cause rare de masse abdominale. Elle touche surtout les jeunes femmes. Elle est d’étiopathogénie encore peu connue, caractérisée par un potentiel malin atténué avec un risque d'extension locale faible et d’évolution métastatique rare. Elle reste de bon pronostic après exérèse chirurgicale complète. Nous rapportons un cas chez une adolescente révélé par une masse épigastrique peu douloureuse. L’échographie abdominale complétée par la tomodensitométrie ont confirmés le diagnostique de tumeur pancréatique. L’étude anatomopathologique et immunohistologique étaient en faveur d'une TPPSP.

## Introduction

La tumeur pseudopapillaire et solide du pancréas est une tumeur rare et une cause rare de masse abdominale. Elle représente 2% des tumeurs exocrines du pancréas. Elle survient presque exclusivement chez l'adolescente ou la jeune femme et elle à un potentiel malin atténué mais de bon pronostique après exérèse chirurgicale [1, 2]. Il n'ya pas de place pour les traitements adjuvants [3]. L'observation d'un nouveau cas de TPPSP est l'occasion de préciser les caractéristiques cliniques et thérapeutiques de cette tumeur.

## Patient et observation

**Figure 1:**
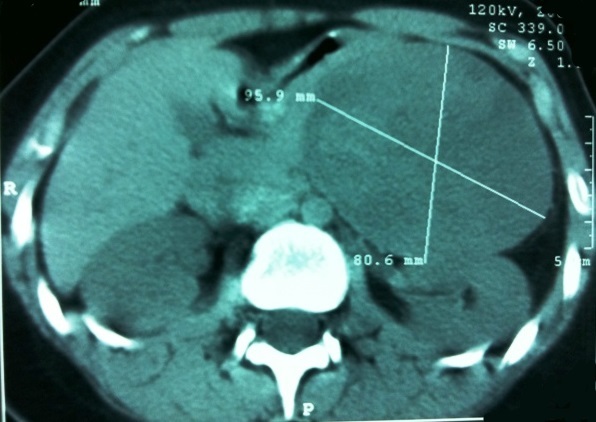
Coupe tomodensitométrique montrant une tumeur arrondie, bien limitée et hétérogène refoulant en bas le rein gauche et en haut l'estomac

**Figure 2:**
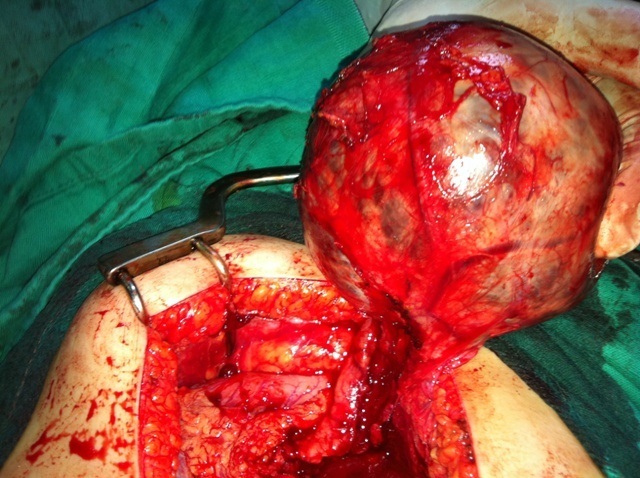
Aspect peropératoire: tumeur aux dépens de la queue du pancréas

Une jeune fille de 16 ans, sans antécédents pathologiques, était hospitalisée pour une douleur abdominale à type de pesanteur, irradiant parfois en postérieur. Cette symptomatologie évoluait depuis 9 mois en s'aggravant, devenant plus accentuée et associée parfois à des vomissements depuis un mois. Elle était apyrétique, en bon état général. La palpation de l'abdomen montrait une masse épigastrique volumineuse, ferme, bien limitée, mobile et légèrement douloureuse. L’échographie abdominale mettait en évidence une masse au niveau du pancréas gauche, bien limitée, d’échostructure hétérogène et de 90 mm de grand axe, sans dilatation du canal de Wirsung. La tomodensitométrie abdominale confirmait le diagnostic et montrait une masse d'environs 80 à 95 mm de diamètre, arrondie, bien limitée, au contact de la queue du pancréas. Elle refoulait l'estomac en avant et le rein gauche en bas. Cette masse était de densité hétérogène, à double composante tissulaire et liquidienne. Il n'y avait pas de lésion hépatique ni adénopathie profonde (Figure 1). La biologie était normale (enzymes pancréatiques,Alphafoetoproteine, CA19-9 ACE). L'exploration chirurgicale, après ouverture de l'arrière cavité des épiploons, trouvait une tumeur ronde et bien limitée siégeant au niveau de la queue du pancréas (Figure 2). Une tumorectomie était réalisée. L’étude anatomopathologique de la pièce opératoire montrait une tumeur encapsulée à surface lisse, pesant 450 g et de 12x10x6 cm de diamètre. Le contenu était kystique avec de vastes remaniements hémorragiques. L’étude immunohistochimique était en faveur d'une (TPPSP). En effet, les cellules tumorales exprimaient la vimentine, l'alpha-1-antitripsyne, la chromogranine, le CD10 et la Béta-Catenine. Les anticorps anti-cytokératine étaient négatifs. Les suites opératoires étaient simples avec un recul d'un an, la patiente étant asymptomatique, sans signe de récidive locale à l’échographie.

## Discussion

La tumeur pseudopapillaire et solide du pancréas est une tumeur rare qui représente moins de 2% des tumeurs pancréatiques et moins de 5% des tumeurs kystiques du pancréas. C'est une tumeur survenant presque exclusivement chez l'adolescente ou la jeune femme (90% entre 20 et 40 ans) [1–3]. En effet, notre patiente est une adolescente de 16 ans. Décrite pour la première fois par FRANTZ en 1959, le terme de tumeur pseudopapillaire et solide a été finalement retenu par l'OMS en 1996 [1, 3]. Sa pathogénie reste peu claire mais il ya deux hypothèses [4]: une possible hormonodépendance vue la prédominance féminine, la présence de récepteurs hormonaux à la surface des cellules tumorales, la croissance tumorale accélérée par l'imprégnation hormonale; une origine embryologique avec une cellule souche totipotente indifférenciée ou germinale qui migrerait et se différencierait secondairement en cellule pancréatique exocrine ou endocrine. Les signes cliniques ne sont pas spécifiques, la symptomatologie est fonction du siège et de la taille de la tumeur. Elle est souvent révélée par une douleur sus ombilicale ou une masse abdominale comme ce fut le cas dans notre observation [3]. Parfois, elle est découverte à l'occasion d'une complication: hémorragie intra tumorale ou rupture tumorale spontanée ou traumatique [3, 5]. La biologie est souvent normale. L'abdomen sans préparation a peu d'intérêt diagnostic. L’échographie, la tomodensitométrie et l'imagerie par résonance magnétique permettent de décrire des formes kystiques, mixtes et des formes solides. Elles montrent habituellement une masse bien limitée, peu vascularisée, se développant préférentiellement dans la région corporéocaudale du pancréas [3]. L’échographie peut montrer des images hypoéchogènes, homogènes ou hétérogènes selon l'importance des zones kystiques. La tomodensitométrie abdominale montre une lésion hétérogène, hypodense, bien limitée avec des remaniements hémorragiques, se rehaussant partiellement en périphérie, parfois des calcifications [3, 5]. L’échographie et le scanner abdominal chez notre patiente montraient une lésion pancréatique à doubles composantes kystique et solide. L'imagerie par résonance magnétique montre des foyers hémorragiques hyperintenses en T1 et T2, entourés d'une capsule souvent hypo-intense sur les séquences pondérées en T2 [3]. Le diagnostic différentiel se pose essentiellement chez l'adulte avec les tumeurs neuroendocrines et les pseudokystes du pancréas [3]. Le traitement des TPPSP est exclusivement chirurgical, allant d'une simple tumorectomie à une pancréatectomie partielle, voire totale selon la topographie de la tumeur. L'exérèse doit être la plus complète possible en évitant les résections trop conservatrices qui exposent au risque d'une récidive tumorale [3]. Notre patiente avait bénéficié d'une résection localisée de la tumeur avec des suites postopératoires simples. L’étude immunohistochimique confirme le diagnostic en éliminant les tumeurs d'une autre nature. Les cellules tumorales expriment la vimentine, l'alpha-1-antitrypsine et la NSE dans 90% des cas. La positivité aux récepteurs de la progestérone est remarquable [3]. Chez notre patiente, les cellules tumorales exprimaient la vimentine et l'alpha-1-antitrypsine, la chromogranine, le CD10 et la Béta-Catenine. Il n'ya pas de place pour les traitements adjuvants. Le pronostic global est bon avec 95% de survie à 5 ans [5].

## Conclusion

La tumeur pseudopapillaire et solide du pancréas doit être évoquée devant toute tumeur kystique du pancréas entrainant une masse épigastrique chez les jeunes femmes. Le diagnostic est confirmé par l’étude immunohistochimique après exérèse chirurgicale, celle-ci doit être la plus complète possible pour éviter une récidive locale. Le pronostic global de cette tumeur reste bon.
